# Factor associated with alcohol use among Lahu and Akha hill tribe youths, northern Thailand

**DOI:** 10.1186/s13011-019-0193-6

**Published:** 2019-01-24

**Authors:** Onnalin Singkorn, Tawatchai Apidechkul, Bukhari Putsa, Sudkhed Detpetukyon, Rachanee Sunsern, Phitnaree Thutsanti, Ratipark Tamornpark, Panupong Upala, Chadaporn Inta

**Affiliations:** 1 School of Health Science, Mae Fah Luang University, Chiang Rai, Thailand; 2 School of Nursing, Mae Fah Luang University, Chiang Rai, Thailand; 3 Center of Excellence for the Hill tribe Health Research, Mae Fah Laung University, Chiang Rai, Chiang Rai Province Thailand

## Abstract

**Background:**

Alcohol use impacts several dimensions, including physical health, mental health, families, and social interactions. This study aimed to estimate the prevalence and to determine the factors associated with alcohol use among Akha and Lahu hill tribe youths in Chiang Rai, Thailand.

**Methods:**

An analytic cross-sectional design was applied to obtain key data on these associations. The study sample was Akha and Lahu hill tribe youths aged 15-24 years who lived in 30 selected hill tribe villages. A questionnaire was developed from an in-depth interview and group discussion and tested for validation and reliability before use. Descriptive statistics were used to demonstrate the general characteristics, and Chi-square test and logistic regression were used to detect associations between variables at α=0.05.

**Results:**

A total of 737 subjects were recruited into the study, of whom 50.0% were Lahu. The average age was 17.9 years, 80.7% were single, 71.1% were Christian, 65.9% graduated secondary school, and 65.7% had their major source of income from their parents. Overall, 17.3% smoked and 45.0% drank alcohol. Among the drinkers, 79.8% drank beer, 61.5% started drinking at an age of 15-19 years, 86.8% had drank for < 5 years, 42.5% were persuaded to drink by their peers, 20.2% suffered an accident after alcohol use, and 17.2% had experienced unsafe sex after drinking alcohol. In the multiple logistic regression, six variables were associated with alcohol use among the Akha and Lahu youths. Males had greater odds of alcohol use than females (OR_adj_ = 3.50, 95% CI = 2.24-5.47). Buddhists had greater odds of alcohol use than Christians (OR_adj_ = 1.88, 95% CI = 1.17-3.04). Participants who were unemployed, employed, and in other categories of occupation had greater odds of alcohol use than those who were students (OR_adj_ = 2.20, 95% CI = 1.23-3.92; OR_adj_ = 6.89, 95% CI = 3.38-13.89; and OR_adj_ = 2.96, 95% CI = 1.01-8.59, respectively). Participants whose fathers were daily wage workers had greater odds of alcohol use (OR_adj_ = 2.89; 95% CI = 1.23-6.79) than those whose parents worked in agriculture, and those whose fathers used alcohol had greater odds of alcohol use than those whose fathers did not use alcohol (OR_adj_ = 2.17, 95% CI = 1.40-3.35). Finally, those who had 6-10 and ≥ 11 close friends living in the same village who used alcohol had greater odds of alcohol use (OR_adj_ = 8.51, 95% CI = 3.10-23.3; and OR_adj_ = 3.84, 95% CI = 1.15-12.77, respectively).

**Conclusion:**

To reduce the initiation of alcohol use among Akha and Lahu youths, public health intervention programs should focus on males who are not attending school and should be implemented for both their family members and peers.

## Background

Alcohol use is one of the most significant determinants of health problems [1, 2]. The World Health Organization (WHO) has reported that hazardous and harmful alcohol use is a major global contributing factor to death, diseases and injuries [3]. The World Health Organization has also reported that globally, 3 million deaths yearly resulted from alcohol use and that 5.1% of global diseases and injuries were attributable to alcohol use, particularly among youths [4]. Alcohol use impacts not only physical and mental health but also family and social problems [5, 6]. The effects are not limited to the users but also affect other people, particularly those who live in close contact with the users, such as family members or community members, through different mechanisms. Accidents are the most frequently reported outcomes among alcohol users, particularly in developing countries such as Thailand, resulting in substantial costs for treatment and therapeutic care for both the users and other impacted persons each year [7, 8]. A number of people have died from accidents mostly caused by alcohol use, especially male youths [9]. The World Health Organization has reported that 6.2% of all male deaths were attributed to alcohol use [10]. Alcohol consumption can impede an entire country’s economic growth due to the large associated expenses in the health care system, particularly in developing countries [11].

Thailand is a developing country that has been ranked 5th worldwide and 1st among the ASEAN countries in alcohol use in 2018, with 30.3% of males and 5.2% of females aged 15 years and above reported to be drinkers [12]. In 2015, approximately 29.5% of Thai youths aged 15-24 years were reported to be alcohol users [13]. In 2018, people aged 15 years and above who lived in the northern region were reported to have the greatest proportion of alcohol use in Thailand [14].

Alcohol use leads to social problems, particularly among youths in northern Thailand [14]. Youths represent a population vulnerable to alcohol abuse [15]. Abuses in these populations are often reported in police and hospital information systems. The World Health Organization reported that 82% of Thai people had experienced adverse effects due to other drinkers at least once within the past 12 months, of which the most vulnerable populations are children, women, and the elderly [16, 17]. The World Health Organization also reported that 28% of the Thai population harmed by alcohol users were impacted by their family members, with children and women being particularly affected because they had less power for protection or negotiation from harm due to alcohol users [18, 19]. Impacts from alcohol use frequently occur in low socioeconomic status communities [20]. Many communities have been identified as low socioeconomic communities in Thailand, including hill tribe communities.

The hill tribe people are a group of people who migrated from southern China to Thailand more than a century ago [21]. Six main groups are living in northern Thailand: the Akha, Lahu, Hmong, Yao, Lisu, and Karen. In 2016, the World Health Organization estimated that 2.5-3.0 million hill tribe people lived in Thailand [22], approximately 65% of whom live under the national poverty line [23]. In the past, hill tribe peoples’ lifestyles depended on their traditional patterns and cultures [24]. Akha and Lahu are the first and second largest hill tribe groups in Chiang Rai province [25]. The Akha and Lahu people are originally from south China and have their own languages, and they are classified into the Tibetto-Burma branch in the Lo-Lo group [26]. In 2017, 673 hill tribe villages were identified in Chiang Rai province [25], of which more than 400 villages were Lahu and Akha. These villages are located in the mountainous and Thai-Myanmar border areas, which are located long distances from cities. Under the management of the Thai government, many Akha and Lahu young children have accessed the Thai educational system [26]. However, schools provide only primary and secondary education, and few people continue their education into high school and university [27].

Alcohol production companies have used several techniques to encourage consumption among new drinkers through television broadcast programs, such as depicting alcohol users as sociable, fighters, talkative, cool, mature, and funny [28]. In villages, the Akha and Lahu have their own lifestyle patterns, particularly in terms of traditional practices related to the use of alcohol for those who still practice their traditional or ancestral religions, including youths [26].

Youths are defined as people aged 15-24 years [29]. They are a major population segment and are the most vulnerable to exposure to alcohol use [30–32]. Given their limited maturity, alcohol use can lead to numerous health problems in this population, including injuries, traffic accidents, violence, suicide, and sexually transmitted diseases [33, 34]. Protecting youths against alcohol use is a significant factor in reducing economic losses and social problems. Therefore, understanding the behavior, culture, patterns, lifestyles, and other factors associated with alcohol use among Akha and Lahu youths is crucial for future public health action. This study aimed to estimate the prevalence and determine the factors associated with alcohol use among Akha and Lahu youths in northern Thailand. The term alcohol use in the study indicates alcohol use disorder according to the American Psychiatric Association definition issued in the 5th edition of The Diagnosis and Statistical Manual of Mental Disorders (DSM-5) [35] which was modified some context on its definition. The purpose was to make most reflection to our study objective which started by question of “Do you use alcohol?”

## Methods

### Study design

An analytic cross-sectional study was applied to estimate the prevalence and determine the behaviors and other factors associated with alcohol use and its impacts among hill tribe Akha and Lahu youths in northern Thailand.

### Study setting

In 2017, there were 321 Akha villages and 278 Lahu villages in Ching Rai [25]. The study was conducted in 30 selected hill tribe villages (15 Akha and 15 Lahu villages) in Chiang Rai Province, Thailand. The targeted villages were selected by a simple random sampling method from lists of the villages of two tribes. The study was conducted between February and November 2017.

### Study population

Lahu and Akha youths aged 15-24 years living in the selected villages in Chiang Rai province, northern Thailand, were the study population. The study populations included Akha and Lahu youths who were attending or not attending school. Those who were not attending school but were working in the city and came back to their village in the evening also met the study criteria.

### Study sample and sample size

People who could identify themselves as Lahu or Akha, were aged 15-24 years old and could provide all essential information in the questionnaires met the eligibility criteria for the study. According to the village’s population information, which was updated by a village headman, 972 youths lived in the 15 selected Lahu villages and 883 youths lived in the 15 selected Akha villages on the start date of the study in 2017.

After calculating the sample size at an accepted error (α) = 0.05, power of the test (1-*β*)=0.8, and prevalence (p) of 23.0% based on a previous study [19] and adding 10.0% to account for any errors or missing data throughout the study process, at least 368 participants were needed from each tribe for the analysis. Finally, a total of 736 participants were required.

### Research instruments

A questionnaire was developed from a literature review and information obtained from an in-depth interview with 20 selected subjects. Ten Akha youths (5 males and 5 females) and 10 Lahu youths (5 males and 5 females) were purposively selected for an in-depth interview regarding alcohol use behaviors. The information obtained from the in-depth interview was used to develop the questionnaires.

The questionnaire consisted of 5 parts. The first part included 18 questions on the general characteristics of the participants, such as age, sex, tribe, and marital status. The second part consisted of questions regarding behavior related to alcohol use, such as “Do you drink alcohol?”, “What kind of alcohol do you most frequently use?”, and “How often do you drink?”. This part included questions that examined both the quality and quantity of alcohol use. In the third part, 10 questions assessed attitudes toward alcohol use. The fourth part included 10 questions focused on detecting the level of knowledge of the impact of alcohol use. In the last part, the participants who used alcohol were asked to provide the level and frequency of their daily alcohol use, which enabled identification of the risk of alcohol use with the alcohol use disorders identification test (AUDITS) [36].

The preliminary constructed questionnaire was tested for validity and reliability by three different methods. The validity was tested by three external experts using the Item Objective Congruence Index (IOC) method [37]. A test-retest method [38] was used to test the reliability among 20 participants (10 Akha and 10 Lahu) in Mae Chan district, Chiang Rai Province. The retest was performed two weeks after the first test, and the reliability coefficient was 0.81. Finally, the questionnaire was piloted in 10 participants who had characteristics similar to the study sample before being fully implemented.

### Data gathering procedure

The lists of Lahu and Akha villages differed according to information from the Hill Tribe Study and Welfare Center, Chiang Rai [23], with a total of 321 Akha villages and 278 Lahu villages in Ching Rai. A multistage random method was used to select the samples. First, the simple random sampling technique was used to select 15 villages from each tribe as the target villages. All selected village headmen were contacted, and verbal approval was obtained before starting the study. A village headman provided a list of their population aged 15-24 years to the researchers. Then, all of the lists were pooled, and the samples were randomly selected. The selected samples were sent back to the village headmen. Prior to the data collection day, all selected samples were introduced to basic essential information about the study through a village broadcast in the morning in the local language.

The questionnaire was provided and completed in a private and confidential room. The questionnaires were mostly completed using a self-administered method after informed consent was obtained from the participants. A few people provided their answers to the questionnaire after the questions were read to them by a researcher assistant who were assigned based on gender matching (i.e., a male interviewer interviewed male participants, and a female interviewer interviewed female participants). The questionnaires were returned to the researchers upon completion and were checked for missing answers before the data collection process was considered complete in each community. All completed questionnaires were kept in a secured location before they were coded with a number and entered into an Excel spreadsheet for analysis. The questionnaire was destroyed once the coding step was completed.

### Statistical analysis

The coded data in the Excel spreadsheet were imported into SPSS (Version 24, 2016, Chicago, IL, USA) for analysis. Descriptive data were used to explain the characteristics of the participants, including frequencies, means, and standard deviations. Characteristics were compared between those who used and did not use alcohol with the Chi-square test. Moreover, specific behaviors regarding alcohol use were evaluated among the users. Logistic regression was used to determine the factors associated with alcohol use among the Lahu and Akha youths at a significance level of alpha = 0.05. In the univariate analysis, associations between one independent variable and one dependent variable were detected at a significance threshold of α=0.05. All variables with significance in the univariate analysis were entered into the multivariate analysis in the same predicted model. Only statistically significant variables were kept in the final model for interpretation.

## Results

A total of 737 participants were recruited into the study from 15 Akha and 15 Lahu villages. Among the selected villages, the closet and farthest from Chiang Rai city were 5 km and 75 km, respectively. Among the participants, 50.0% were Lahu, 50.3% were female, 71.8% were aged 15-19 years (average age = 17.9 years), and 80.7% were single. Five hundred and twenty-four were Christians, and most had graduated from secondary school (65.9%) at the date of the interview. The major source of income was their parents (65.7%), 73.1% had an income only on school days, and 83.9% had an income ≤5000 baht per month (Table 1).

**Table 1:** Comparison of general characteristics by alcohol use and none groups

Characteristics	Total (%)	Alcohol use		χ^2^	*p*-value
		No (%)	Yes (%)		
Total	737(100.0)	405 (55.0)	332 (45.0)	N/A	N/A
Tribe					
Lahu	369 (50.0)	212 (57.4)	157 (42.6)	2.06	0.151
Akha	368 (50.0)	193 (52.4)	175 (47.6)		
Sex					
Male	366 (49.7)	135 (36.9)	231 (63.1)	95.87	< 0.001^a^
Female	371 (50.3)	270 (72.8)	101 (27.2)		
Age (years)					
15–19	529 (71.8)	317 (59.9)	212 (40.1)	18.71	< 0.001^a^
20-24	208 (28.2)	88 (42.3)	120 (57.7)		
Marital status					
Single	595 (80.7)	329 (55.3)	266 (44.7)	0.47	0.790
Married	125 (17.0)	68 (54.4)	57 (45.6)		
other	17 (2.3)	8 (47.1)	9 (52.9)		
Religion					
Buddhist	213 (28.9)	96 (45.1)	117 (54.9)	11.81	0.001^a^
Christianity	524 (71.1)	309 (59.0)	215 (41.0)		
Education					
Illiterate	53 (7.2)	23 (43.4)	30 (56.6)	7.83	0.049^a^
Primary school	84 (11.4)	40 (47.6)	44 (52.4)		
Secondary school	486 (65.9)	284 (58.4)	202 (41.6)		
Vocational school	114 (14.6)	58 (50.9)	56 (49.1)		
Occupation					
Student	417 (56.6)	276 (66.2)	141 (33.8)	88.88	< 0.001^a^
Unemployed	133 (18.0)	60 (45.1)	73 (54.9)		
Agriculture	47 (6.4)	28 (59.6)	19 (40.4)		
Employed	114 (15.5)	27 (23.7)	87 (76.3)		
Other	26 (3.5)	14 (53.8)	12 (46.1)		
Major source of income					
Work	161 (21.8)	40 (31.4)	121 (68.6)	78.15	< 0.001^a^
Parents	484 (65.7)	300 (61.5)	184 (38.5)		
Couple	53 (7.2)	36 (67.9)	17 (32.1)		
Other	39 (5.3)	29 (67.1)	10 (32.9)		
Characteristics of income					
Regularly	198 (26.9)	108 (54.5)	90 (45.5)	0.01	0.892
Only school days	539 (73.1)	297 (55.1)	242 (44.9)		
Income per month (baht)					
≤ 5000	618 (83.9)	370 (59.9)	248 (40.1)	28.27	< 0.001^a^
5001-10,000	101 (13.7)	30 (29.7)	71 (70.3)		
≥ 10,001	18 (2.4)	5 (27.8)	13 (72.2)		
Living with					
Alone	40 (5.4)	26 (65.0)	14 (35.0)	5.25	0.262
Parents	493 (66.9)	264 (53.5)	229(46.5)		
Father	34 (4.6)	15 (44.1)	19 (55.9)		
Mother	48 (6.5)	26 (54.2)	22 (45.8)		
Other	122 (16.6)	74 (60.7)	48 (39.3)		
Number of family member (persons)					
≤ 5	385(52.2)	201 (52.2)	184 (47.8)	2.60	0.272
6-10	341 (46.3)	197 (57.8)	144 (42.2)		
> 10	11 (1.5)	7 (63.6)	4 (36.4)		
Father’s education					
Illiterate	486 (65.9)	273 (56.2)	213 (43.8)	1.99	0.369
Primary school	101 (13.7)	49 (48.5)	52 (51.5)		
Secondary school	150 (20.4)	83 (55.3)	67 (44.7)		
Father’s occupation					
Agriculture	345 (46.8)	211 (60.0)	134 (40.0)	14.93	0.001^a^
Daily employed	310 (42.1)	161 (51.9)	149 (48.1)		
Small grocery owner	36 (4.9)	17 (47.2)	19 (52.8)		
Unemployed	46 (6.2)	16 (34.8)	30 (65.2)		
Alcohol use behavior of father					
No	332 (45.0)	232 (69.9)	100 (30.1)	54.37	< 0.001^a^
Yes	405 (55.0)	173 (42.7)	232 (57.3)		
Mother’s education					
Illiterate	533 (72.3)	302 (56.7)	231 (43.3)	2.44	0.295
Primary school	96 (13.0)	47 (49.0	49 (51.0)		
Secondary school	108 (14.7)	56 (51.9)	52 (48.1)		
Mother’s occupation					
Agriculture	378 (51.9)	224 (59.3)	154 (40.7)	5.91	0.115
Daily employed	260 (35.2)	131 (50.4)	129 (49.6)		
Small grocery owner	44 (5.6)	23 (52.2)	21 (47.7)		
Unemployed	55 (7.3)	27 (49.1)	28 (50.9)		
Alcohol use behavior of mother					
No	598 (81.1)	344 (57.5)	254 (42.5)	8.47	0.003^a^
Yes	139 (18.9)	61 (43.9)	78 (56.1)		
Having conflict with parents					
No	206 (28.0)	111 (53.9)	95 (46.1)	0.18	0.911
Sometime	501 (68.0)	278 (55.5)	223 (44.5)		
Often	30 (4.0)	16 (53.3)	14 (46.7)		
Parents’ conflicting					
No	184 (24.9)	100 (54.3)	84 (45.7)	0.13	0.889
Sometime	519 (70.4)	285 (54.9)	234 (45.1)		
Often	34 (4.6)	20 (57.6)	14 (42.4)		
Having close friend lived in the same village					
No	93 (12.3)	60 (64.5)	33 (35.5)	3.93	0.047^a^
Yes	644 (87.7)	345 (53.6)	299 (46.4)		
Number of close friend lived the same village and used alcohol (person)					
≤ 5	545 (84.3)	330 (60.5)	215 (39.5)	67.51	< 0.001^a^
6-10	69 (10.9)	8 (11.6)	61 (88.4)		
> 10	30 (4.8)	7 (23.3)	23 (76.7)		
Having close friend outside the village					
No	210 (29.0)	130 (61.9)	80 (38.1)	5.73	0.016^a^
Yes	527 (71.0)	275 (52.2)	252 (47.8)		
Number of close friend outside the village who used alcohol (persons)					
≤ 5	306 (58.1)	186 (60.8)	120 (39.2)	20.38	< 0.001^a^
6 - 10	126 (23.9)	54 (42.9)	72 (57.1)		
> 10	95 (18.0)	35 (36.8)	60 (63.2)		
Smoking					
No	610 (82.7)	384 (62.9)	226 (37.1)	91.48	< 0.001^a^
Yes	127 (17.3)	21 (16.5)	106 (83.5)		
Knowledge regarding the impact on alcohol use					
Low	323 (43.8)	158 (48.9)	165 (51.1)	10.93	0.004^a^
Moderate	372 (50.4)	223 (60.0)	149 (40.0)		
High	42 (5.8)	24 (57.1)	18 (42.9)		
Attitude regarding the impact on alcohol use					
Low	12 (1.6)	4 (33.3)	8 (66.7)	41.18	< 0.001^a^
Moderate	372 (50.5)	164 (44.0)	208 (56.0)		
High	353 (47.9)	237 (67.1)	116 (32.9)		

^a^Significant level at α=0.05

Most of the participants lived with their parents (66.9%), and more than half of their parents were illiterate (65.9% of the fathers and 72.3% of the mothers). A total of 88.9% of the fathers and 87.1% of the mothers of the participants were working as farmers and daily wage employees. Additionally, 55.0% of the fathers and 18.9.0% of the mothers used alcohol. A total of 72.0% had ever had a conflict with their parents, and 75.0% had witnessed a conflict between their parents (Table 1).

A large proportion of the participants had a close friend living in the same village (87.7%). All participants reported having a close friend inside or outside the village who used alcohol. A total of 84.3% had ≤5 close friends living in same village who used alcohol, and 58.1% had ≤5 close friends living outside the village who used alcohol. A total of 17.3% smoked, 52.1% were in the low and moderate attitude level group regarding the impact of alcohol use, and 94.2% had low and moderate levels of knowledge about the impact of alcohol use (Table 1).

Some significant differences in characteristics were found between the alcohol users and nonusers. A greater proportion of the alcohol users were males than females (*p*-value< 0.001). Participants aged 20-24 years had a greater proportion of alcohol users than those aged 15-19 years (*p*-value< 0.001), and those who were Buddhists had a greater proportion of alcohol users than those who were Christians (*p*-value = 0.001). Those with a low education level had a greater proportion of alcohol users than those with a higher education level (p-value = 0.049). Participants with an income from work had a greater proportion of alcohol users than those who did not (p-value< 0.001), and those with an income from their own work had a greater proportion of alcohol users than those who were dependent on another’s income (p-value≤0.001). Moreover, those with a higher income had a greater proportion of alcohol users than those with a lower income (p-value = 0.001). Participants whose parents drank alcohol and were unemployed tended to use alcohol more often than those whose parents did not use alcohol and were employed. Those with a large number of close friends who used alcohol within and outside the village had a higher proportion of alcohol users than those who did not. Participants with poor knowledge and attitudes regarding the impact of alcohol use were more likely to be alcohol users than those with good knowledge and attitudes concerning the impact of alcohol use (Table 1).

Among the 332 participants (45.0%) who reported alcohol use, two-thirds started their alcohol use at 15-19 years of age (61.5%), most had less than 5 years of experience with alcohol consumption (86.8%), and 79.8% started drinking with beer. The most common reason they provided for drinking alcohol was persuasion from their peers (42.5%) and curiosity (32.3%). A total of 78.6% of the participants had used alcohol within the past 12 months, 70.8% had used alcohol within the past 30 days, 50.9% had used alcohol within the past week, and 8.2% used alcohol every day. One-fourth used drugs while drinking alcohol (25.6%). The preferred place for drinking alcohol was their peers’ houses (56.9%), and 41.3% reported that they used their own wages for alcohol expenses. One-fifth (20.5%) had suffered an accident or injury after drinking alcohol, and 17.2% had an unsafe sexual experience with people who were not regular partners. Only 20.2% reported that they would no longer use alcohol in the future (Table 2).

**Table 2:** Characteristics of alcohol users

Characteristics	Number	Percent
Total	332	100.00
Initiation of drinking at (years)		
≤ 14	111	33.4
15-19	204	61.5
20-24	17	5.1
*Max = 24)*	*(Mean = 15.34, SD = 2.55, Min = 11,*	
Lifetime use of alcohol (years)		
< 5	288	86.8
5-10	40	12.0
> 10	4	1.2
Type of first alcohol beverage used		
Beer	265	79.8
Local whisky	36	10.8
Commercial whisky	12	3.6
Local modified whisky	3	0.9
Wine	13	3.9
Juice mixed alcohol	3	0.9
Major reason of initiation use alcohol		
Peer persuaded	141	42.5
Engage to know	107	32.3
Social engagement	30	9.0
To have a friend	11	3.3
Reduce the stress	21	6.3
Relief from work	16	4.8
Motivated by advertising	6	1.8
Use alcohol in the past 12 months		
No	71	21.4
VYes	261	78.6
Use alcohol in the past 30 days		
No	97	29.2
Yes	235	70.8
Use alcohol in the past week		
No	163	49.1
Yes	169	50.9
Frequency in use alcohol in the past week		
7 days/week	14	8.2
5-6 days/week	16	9.5
3-4 days/week	52	30.8
1-2 day/week	87	51.5
Drug use during drinking alcohol		
No	247	74.4
Yes	85	25.6
Type of drug		
Cigarette	83	97.6
Methamphetamine	2	2.4
Drinking with		
Alone	16	4.8
Male friends	186	56.0
Female friends	63	18.9
Both male and female friends	44	13.3
Co-workers	20	6.0
Other	3	0.9
Place for drinking		
Owner house	46	13.9
Peer’s house	189	56.9
Apartment	11	3.3
Office	20	6.0
Club	28	8.4
Other	38	11.5
Source of money for alcohol expenses		
Parents	61	18.4
Personal wage	137	41.3
Friend	93	28.0
Other	41	12.3
Expenses for alcohol per month (baht) *Mean = 585.27, SD = 842.58, Min = 0, Max = 5000*		
Haggling experience after alcohol use		
No	285	85.8
Yes	47	14.2
Having traffic accident		
No	264	79.5
Yes	68	20.5
Having sex with people who were not regular partner with unused condom		
No	275	82.8
Yes	57	17.2
Prospective decision in future alcohol use		
Not sure	145	43.7
Reduce the frequency	104	31.3
Will not use	67	20.2
No change	11	3.3
Increase amount and frequency	5	1.5

When the alcohol users were classified by the outcomes of the alcohol use disorders identification test (AUDITS), 35.5% were risky drinkers, 6.2% were harmful drinkers, and 10.9% were addicted drinkers (Table 3).

**Table 3:** Level of the risk from alcohol use classified by the alcohol use disorders identification test (AUDITS)

Level	Number	Percent
Low risk drinker	157	47.3
Risky drinker	118	35.5
Harm drinker	21	6.3
Addicted drinker	36	10.9

In the simple logistic regression, 12 variables were associated with alcohol use among the Lahu and Akha youths: sex, age, religion, occupation, income, father’s education, father’s occupation, mother’s occupation, alcohol use by father, alcohol use by mother, number of close friends inside the village who used alcohol, and number of close friends outside the village who used alcohol (Table 4).

**Table 4:** Univariate and multivariate analyses of factors associated with alcohol use among the Akha and Lahu youths

Characteristics	Crude OR	95%CI	p-value	Adjusted OR	95%CI	*p*-value
Tribe						
Lahu	1.00					
Akha	1.24	0.93-1.66	0.152	N/S	–	–
Sex						
Female	1.00			1.00		
Male	4.59	3.36-6.28	< 0.001^a^	3.50	2.24-5.47	< 0.001^a^
Age (year)						
15-19	1.00					
20-24	2.01	1.45-2.78	< 0.001^a^	N/S	–	–
Religion						
Christianity	1.00			1.00		
Buddhist	1.75	1.27-2.41	0.001^a^	1.88	1.17-3.04	0.009^a^
Marital status						
Single	1.00					
Married	1.03	0.70-1.52	0.864	N/S	–	–
Other	1.39	0.53-3.65	0.506	N/S	–	–
Education						
Illiterate	1.35	0.70-2.60	0.369	N/S	–	–
Primary school	1.14	0.65-2.00	0.650	N/S	–	–
Secondary school	0.74	0.49-1.11	0.146	N/S	–	–
Vocational school	1.00					
Occupation						
Student	1.00			1.00		
Unemployed	2.41	1.62-3.59	< 0.001^a^	2.20	1.23-3.92	0.008^a^
Agriculture	1.32	0.71-2.45	0.373	1.16	0.44-3.07	0.757
Employed	6.28	3.90-10.12	< 0.001^a^	6.85	3.38-13.86	< 0.001^a^
Other	1.67	0.75-3.71	0.207	2.96	1.01-8.59	0.046^a^
Income (baht)						
≤5000	1.00					
5001-10,000	3.49	2.21-5.52	< 0.001^a^	N/S	–	–
≥10,001	3.83	1.35-10.89	0.012^a^	N/S	–	–
Number of family member (persons)						
≤5	1.00					
6-10	0.79	0.59-1.07	0.123	N/S	–	–
≥11	0.62	0.18-2.15	0.449	N/S	–	–
Father’s education						
Illiterate	0.35	0.13-0.92	0.033^a^	N/S	–	–
Primary school	0.42	0.15-1.18	0.101	N/S	–	–
Secondary school	1.00					
Mother’s education						
Illiterate	0.34	0.10-1.09	0.070	N/S	–	–
Primary school	0.42	0.12-1.45	0.171	N/S	–	–
Secondary school	1.00					
Father’s occupation						
Agriculture	1.00			1.00		
Daily employed	1.50	1.05-2.13	0.025^a^	0.97	0.60-1.56	0.903
Small grocery owner	1.67	0.83-3.37	0.148	1.33	0.55-3.19	0.519
Unemployed	2.81	1.46-5.41	0.002^a^	2.89	1.23-6.79	0.015^a^
Mother’s occupation						
Agriculture	1.00					
Daily employed	1.27	0.90-1.80	0.171	N/S	–	–
Small grocery owner	1.54	0.76-3.14	0.229	N/S	–	–
Unemployed	2.06	1.09-3.89	0.026^a^	N/S	–	–
Alcohol use of father						
No	1.00			1.00		
Yes	2.93	2.08-4.11	< 0.001^a^	2.17	1.40-3.35	< 0.001^a^
Alcohol use of mother						
No	1.00					
Yes	1.78	1.19-2.68	0.005^a^	N/S	–	–
Having conflict with parents						
No	1.00					
Sometime	0.93	0.67-1.29	0.669	N/S	–	–
Often	1.01	0.47-2.18	0.973	N/S	–	–
Parents’ conflicting						
No	1.00					
Sometime	0.96	0.68-1.35	0.967	N/S	–	–
Often	0.87	0.41-1.84	0.870	N/S	–	–
Number of closed friend from same village who used alcohol (person)						
≤5	1.00			1.00		
6-10	11.28	5.29-24.04	< 0.001^a^	8.51	3.10-23.37	< 0.001^a^
≥11	4.86	2.05-11.53	< 0.001^a^	3.84	1.15-12.77	0.028^a^
Number of closed friend from outside the village who used alcohol (persons)						
≤5	1.00					
6-10	3.98	2.01-7.88	< 0.001^a^	N/S	–	–
≥11	5.18	2.51-10.67	< 0.001^a^	N/S	–	–
Knowledge regarding the impact on alcohol use						
Low	1.39	0.48-2.36	0.231	N/S	–	–
Moderate	1.22	0.77-2.18	0.304	N/S	–	–
High	1.00					
Attitude regarding the impact on alcohol use						
Low	1.42	0.88-1.73	0.451	N/S	–	–
Moderate	1.09	0.61-1.42	0.360	N/S	–	–
High	1.00					

^a^Significant level at α=0.05; N/S = Non-significant

In the multiple logistic regression analysis, six variables were associated with alcohol use among the Akha and Lahu youths. Males had greater odds of alcohol use than females (OR_adj_ = 3.50, 95% CI = 2.24-5.47). Buddhists had greater odds of alcohol use than Christians (OR_adj_ = 1.88, 95% CI = 1.17-3.04). Participants who were unemployed, employed, and in other categories of occupation had greater odds of alcohol use than students (OR_adj_ = 2.20, 95% CI = 1.23-3.92; OR_adj_ = 6.89, 95% CI = 3.38-13.89; and OR_adj_ = 2.96, 95% CI = 1.01-8.59, respectively). Participants whose fathers were daily wage workers had greater odds of alcohol use (OR_adj_ = 2.89, 95% CI = 1.23-6.79) than those whose parents worked in agriculture, and those whose fathers used alcohol had greater odds of alcohol use than those whose fathers did not use alcohol (OR_adj_ = 2.17, 95% CI = 1.40-3.35). Finally, those who had 6-10 and ≥ 11 close friends living in the same village who used alcohol had 8.51 (95% CI = 3.10-23.37) and 3.84 (95% CI = 1.15-12.77) greater odds of alcohol use, respectively (Table 4).

## Discussion

Akha and Lahu youths are vulnerable to alcohol use, with a prevalence of alcohol users of 45.0%. Males had a greater proportion of alcohol users than females, but no difference was found between tribes. The majority initiated their alcohol use between 15 and 19 years of age with beer. The major reason that they initiated alcohol consumption was persuasion from their peers and curiosity. A large proportion was classified as regular drinkers, and some individuals used drugs while drinking alcohol. One-fifth had suffered an accident or injury after drinking alcohol, and some had experienced unsafe sexual intercourse with a non-regular partner and had been in an argument. Several factors were associated with the initiation of alcohol use among Akha and Lahu youths, including religion, occupation, parents’ occupations, parents’ alcohol use behaviors, and the number of their peers who used alcohol.

This study had a few limitations. One participant could not identify their age; because no other evidence could be used for identification, he was excluded from the study. Recall bias is always a significant factor in a cross-sectional study. However, alcohol use behavior is a common occurrence in individuals, and the answers to questions regarding frequent behaviors do not tend to deviate from the truth.

In our study, we found that the prevalence of alcohol use among the Akha and Lahu youths was very high (45.0%) compared to the average for Thai youths, which was reported to be 27.9% in 2015 [39]. The prevalence in this study was also higher than the prevalence of alcohol use among youths living in northern Thailand, which was reported to be 28.3% in 2017 [40]. No significant difference in the prevalence was found between the Akha and Lahu youths. This result coincides with that of the study of Banks et al. among American adolescents aged 12-18 years in 2017, which found no difference in expectancies and consumption among different races [41].

Cigarettes are the major drug used while drinking alcohol among the Akha and Lahu youths, particularly by males. This result is similar to that of the report of Karl et al. among 65,528 Korean students in 2016, which found that 13.3% were concurrent lifetime smokers and drinkers, with males having a greater proportion than females [42]. Karl et al. also reported that factors such as male sex, higher education, lower paternal and maternal education levels, lower socioeconomic status, and living in rural areas were associated with alcohol use [42]. A study in California among youths regarding simultaneous alcohol and marijuana use found that contexts with no other underage drinkers were associated with a 99.0% lower risk of simultaneous use [43]. Moreover, the data analysis from the 2011-2014 National Survey on Drug Use and Health in the United States with a total of 14,667 participants found that for the majority of alcohol users among youths, females reported using only alcohol, whereas males were more likely to be concurrent alcohol, marijuana, and cigarette users [44].

In this study, we found that the earliest age of initiation of alcohol use was 12 years old among the Akha and Lahu youths. This age is less than that of a study in Slovakia that found that the initiation age of alcohol use among the Slovakian youths was ≤13 years old and that males had a greater proportion of alcohol users than females [45]. Moreover, use of alcohol from an earlier age tends to have consequences. Doran et al. reported that the onset of first alcohol use was strongly predictive of earlier sexual intercourse for both males and females, with the effects of drinking most pronounced for females during early adolescence [46]. Moreover, Donoghue et al. found that an age at first alcohol use younger than 15 years was associated with cigarette use, a lower quality of life, alcohol-related health and social consequences, and evidence of an alcohol use disorder among youth populations in England [47].

We also found that the Akha and Lahu youths favored beer and local whisky and that the initiating beverage for most of them was beer. Males preferred drinking local whisky over other beverages. Drinking alcohol was symbolic because it showed strong physical health and readiness to be a community leader [48]. In terms of the prevalence of alcohol use, we also found that the older age category had a higher prevalence than the younger age categories.

The reasons for alcohol use among the Akha and Lahu youths were persuasion by their peers and curiosity. In 2016, a study in Canada among adolescents aged 11-20 years old found a link between the use of social networking sites among youths and alcohol use [49]. A longitudinal study in Australian youths that examined the association between the density of alcohol outlets and alcohol use among the 2835 youths reported that the participants’ perceptions of alcohol availability, alcohol use by friends and a higher alcohol density were associated with alcohol consumption by youths at an early age [50]. In our study, we found that having friends who used alcohol inside and outside the village was significantly associated with alcohol use in the Akha and Lahu youths. The number of close friends who used alcohol had a great impact on the initiation of alcohol use among Akha and Lahu youths. This finding coincides with that of the study of Nuria et al., who reported that the main reason for engaging in alcohol use among Spanish youths was enhancing their social relationships, which acted as a mechanism to normalize intensive alcohol consumption [51].

Several factors were related to alcohol use among the Akha and Lahu youths in this study, including religion, occupation, parents’ occupations, parents’ alcohol use behaviors and the number of their peers who used alcohol. The proportions of Christians among the Akha and Lahu were similar (69.3, and 72.8%, respectively). However, in the multivariate analysis, Buddhists had 1.88 greater odds of alcohol use than Christians. Many religious rituals are related to alcohol use among the Akha and Lahu [48], which may impact the initiation of alcohol use, particularly in males. However, several studies have reported that religion played a role as a protective factor against the initiation of alcohol use [52, 53].

Our study found that having more close friends led to greater odds of alcohol use among Akha and Lahu youths; in particular, having close friends who used alcohol made the participants more likely to use alcohol. In the in-depth interview stage, some youths said that loneliness and stress were a cause of initiation of alcohol use among hill tribe youths. Michael conducted a study among UK youths in 2017 and found that being female and experiencing loneliness were linked to greater risks of alcohol use within the past 30 days than the risk for males [54]. Moreover, a study by Aranza et al. reported that restraint stress significantly increased the rate of alcohol intake and preference in females but decreased alcohol intake and preference in males [55]. However, in our study, we found that males tended to use alcohol more than females, which might be due to social norms. Most hill tribe people have fewer males than females [48]. Thus, increased acceptance of male but not female alcohol use was not our expectation. Eric et al. reported that perceived injunctive and descriptive norms for male youths were associated with all alcohol drinking outcomes, including past year use, past month frequency, past month quantity, and peak drinking [56]. This result was supported by a study in China among minorities in Yunnan Province, which is located close to northern Thailand; that study found that living in a drinking environment and particularly having close friends who used alcohol had direct effects on alcohol use [57].

We found that the Akha and Lahu youths with occupations and those who earned higher incomes had greater odds of engaging in alcohol use than those who were students and those who had no income. This result coincides with that of a study conducted in 10 different countries including Thailand that assessed the associations of different characteristics between those who did and did not use alcohol and found that income was significantly associated with alcohol use [58]. Yi et al. also supported the finding that having an income was a significant factor for alcohol use among university students in ASEAN countries [59].

Father’s alcohol use behavior and occupation were significantly associated with alcohol use among their children in Akha and Lahu youths. He et al. [57] also reported that the father’s alcohol use behavior impacted alcohol use by their children. A study in the Netherlands supported an association of father’s alcohol use as a direct path for alcohol use in their high school-aged children [60]. Moreno et al. [61] also reported that among Hispanics, greater social support from and greater negative interactions with the father figure were more predictive of earlier onset of alcohol use, whereas greater social support from the mother figure was more predictive of a later alcohol onset. Even in a study conducted in Thailand, family members’ alcohol use, particularly parents’ alcohol use, was a significant associated factor for alcohol use in their high school-aged children [62].

Thailand has several policies regarding alcohol control and prevention through several mechanisms such as restriction on the hours of sale, raising the minimum age limit of purchase, restricting proximities of alcohol outlets from schools [63, 64]. Even though Thailand has a good policy and has been implemented for several years to control alcohol, some policies have not successful for its implementation such as failed to control the number of alcohol outlets, increasing tax rates, and compliance to age and time restrictions [65]. Moreover, these policies are not too much effective implementation in the remote hill tribe villages due to limit of number of government office to monitor regarding the regulations.

## Conclusion

Alcohol use is a public health problem in Akha and Lahu youths. Our study shows that the prevalence of alcohol use in hill tribe youths is also very high and that the initiation age of alcohol use is as early as 10 years old. The major factors associated with alcohol use among hill tribe youths include personal characteristics, parent’s behaviors and peers’ behaviors, including sex, religion, occupation, father’s occupation, and father’s and close friends’ alcohol use behaviors. Public health interventions focused on improving personal and social skills to avoid alcohol use among Akha and Lahu youths should be implemented, particularly for males.

## Abbreviations

ASEAN: Association of Southeast Asian Nations
AUDITS: The alcohol use disorders identification test
IOC: Item Objective Congruence Index
OR: Odds ratio
WHO: World Health Organization
